# 

**Published:** 1878-06

**Authors:** 


### CONTENTS.

### COMMUNICATIONS.

Interglobular Spaces.—*By D. C. Hawxhurst*.............................. 221
Oral Secretions.—*By G. S. Shattuck, D. D. S*........... .................... 225
Analysis of Normal Saliva.—*By B. F. Miller, D. D. S*................ 229

### SELECTIONS.

Surgery of the Face.—*By Francis Mason, F. R. C. S*.... ......... ......... 233
Practical Remarks on the Doses of Medicine.—*By R. H. Dalton, M. D* 240
Microscopic Preparations.................................... .................... 247
Surgery............. ............................ ................................. 250
Treatment of Serous Effusions by Limitation of Fluid.................... 252

### PROCEEDINGS OF SOCIETIES.

Proceedings of the Ohio Dental College Association ........................ 254

### EDITORIAL.

National Association............................................................ 257
Wisconsin State Dental Society............................................ 259
A New and Good Move.......................................................... 260
Southern Dental Association............................. .... ........... 261
Connecticut Valley Dental Society....................... .............. .... 261
American Dental Association.................................. ........... 263
A New Dental College............................................................ 263

